# Efficacy and safety of platelet-rich plasma combined with Tai Chi for knee osteoarthritis: study protocol for a placebo-controlled randomized trial

**DOI:** 10.1186/s13018-023-04372-6

**Published:** 2023-11-21

**Authors:** Kun Yang, Yuwu Ding, Haichen Xu, Minghui Lai, Xueping Xu, Xiaoming Yu, Qian Deng, Liming Jiang

**Affiliations:** 1 Department of Rehabilitation, Seventh People’s Hospital of Shanghai University of Traditional Chinese Medicine, Shanghai, China; 2 School of Health and Nursing, Wuxi Taihu University, Wuxi, China

**Keywords:** Osteoarthritis, Knee, Platelet-rich plasma, Tai Chi

## Abstract

**Background:**

No definitive treatment methods of curative for knee osteoarthritis (KOA). The combined therapies that into account both the biochemical and biomechanical may provide potential opportunities for treat KOA, and previous studies have demonstrated that the platelet-rich plasma of intra-articular injection (IAI-PRP) and exercise treatments afford more benefits than do their corresponding monotherapies. The absence of a specific exercise plan and detailed explanation renders the aforementioned study results questionable. Furthermore, Tai Chi (TC) with moderate-intensity, whole body movements and good adherence may prove to be more effective for treating KOA. However, few studies examined the effectiveness and safety of combined IAI-PRP and TC for KOA.

**Methods:**

This study protocol will be a placebo-controlled, assessor-blinded randomized trial involving 12-week intervention and 1-year follow-up. The stratified randomization will be used to randomly assign the 212 participants to four groups: group A (placebo IAI); group B (PRP IAI); group C (TC and placebo IAI); group D (TC and PRP IAI). Injection will be performed once a week, three consecutive times as a course, after a week of rest to continue the next course, a total of 3 courses (12 week). Additionally, the TC interventions will be carried out 3 days per week for a total of 12 weeks. The primary outcome measures will include the efficacy (Western Ontario and McMaster Universities Osteoarthritis Index), acceptability and safety of these interventions. The secondary outcome measures will include physical function (Timed Up and Go test), walking function (Gait Analysis), inflammatory factor levels (e.g., Interleukin-1 β, interleukin-6, vascular endothelial growth factor), quality of life (36-Item Short Form Health Survey), volume of patellofemoral cartilage and effusion-synovitis (MRI). Two-way of variance with repeated measures will be applied to examine the main effects of the group and the time factor and group-time interaction effects for all outcome measures.

**Discussion:**

This trial will be first one to propose an integrated scheme combing IAI-PRP and TC for treatment of KOA, based on the consideration of the biochemical and biomechanical pathogenesis of KOA. These results of the study will provide evidence with high quality for integrated IAI-PRP and TC to treatment KOA.

*Trial Registration* Chinese Clinical Trial Registry ChiCTR2300067559. Registered on 11 January 2023.

## Background

Knee osteoarthritis (KOA) is a chronically orthopedic disease with cartilage degeneration and knee function limitations, which manifests as pain, swelling, stiffness and dysfunction, and severely restricts daily activities and mobility [1, 2]. The epidemiological study reported that the pooled global prevalence of KOA is 22.9% in people aged 40 and over [3]. More importantly, the incidence of KOA has continued to grow over the past few decades, in line with the global phenomenon of longevity and aging populations [4]. There is an important effort worldwide to establish therapeutic strategies to modify the natural history of KOA [2, 5]. However, no definitive methods of curative or reverse degeneration process for KOA, which meaning that treatment focuses on ways to treat patient symptoms and slow the progression of the degenerative process.

The challenge of treatment methods lies in the complexity and unclearly of pathological mechanisms involved in KOA [6]. Although the exact pathophysiology mechanism is still unclear, the biochemical and biomechanical are recognized as the key mechanism to the occurrence and development of KOA [7–9]. In aspect of biochemical, the inflammatory factors, abnormal chondrocyte apoptosis, and extracellular degradation are closely associated with the occurrence and development of KOA [10, 11]. In aspect of biomechanical, the atrophy of quadriceps femoris, lower-extremity malalignment, and abnormal stress distribution of the knee joint are associated with pain aggravation and cartilage loss progression [9, 12]. It should be emphasized that the biochemical mediated processes and biomechanical factors are coupled in the KOA development, which probably causing a vicious circle of cause and effect [7]. As a result, the concept of combined therapies has been proposed to treat KOA that take into account both the two aspects, an example commonly seen is the combination of intra-articular injection and exercise training [13].

Intra-articular injection (IAI) is commonly used as a conservative treatment for KOA, as it has the potential to induce regenerative changes in tissue structure and alleviate symptoms [5, 14–16]. Among of some injectants, the platelet-rich plasma (PRP) is the most promising alternative during the pain alleviation, restoration of the functional capacity and potential tissue repairment for KOA, due to this contains large amounts of growth factors and anti-inflammatory cytokines [6, 17]. Several previous studies have reported that the IAI-PRP and exercise treatments afford more benefits than do their corresponding monotherapies in patients with KOA [18, 19]. However, the absence of a specific exercise plan and detailed explanation renders the aforementioned study results questionable. Such as, Kaszynski et al. [18] and Paterson et al. [20] permitted patients with KOA to participate in weight-bearing and progressive physical activities within tolerable limits, but they did not provide a comprehensive and specific exercise plan with detailed explanations; while Raeissadat et al. [19] provided the description of multi-angle isometric strengthening exercises for the knee muscles, it is important to note that knee extensor strength is just one aspect influencing the progression of KOA. In contrast, whole body movements that involve lower limb motion chains, center of gravity transfer, and posture control may prove to be more effective for treating KOA. As a result, it is crucial to develop a comprehensive and reproducible clinical protocol to reassess the clinical benefits of combining IAI-PRP and exercise.

Tai Chi (TC), as a moderate-intensity exercise training, was recommended by American College of Rheumatology for KOA management [21–23]. A systematic review reported that TC was an effective and safety way for relieve pain, improve muscle strength, adjust lower-extremity alignment, increase joint stability, and balance control for the patients of KOA [22]. Furthermore, few patients have complained of the side-effects after receiving TC exercise [24]. However, few studies examined the effectiveness and safety of combined IAI-PRP and TC for KOA. Furthermore, the differences of efficacy between IAI-PRP and TC in the KOA treatment were not clarified in the previous study.

Therefore, the placebo-controlled, assessor-blinded randomized trial will be design in the present protocol, which will include group A (IAI-placebo), group B (IAI-PRP), group C (TC + IAI-placebo), and group D (TC + IAI-PRP). This protocol will allow to be achieve the study purpose as follows:

Aim 1: To examine the effectiveness and safety of combined IAI-PRP and TC for KOA treatment by comparing the group D vs B or C.

Aim 2: To compare the differences of efficacy between the IAI-PRP and TC in the KOA treatment by comparing the group B and C.

Aim 3: To provide high-quality evidence of PRP for KOA treatment by comparing the group B and A.

## Methods/Design

### Study design

**Fig. 1:**
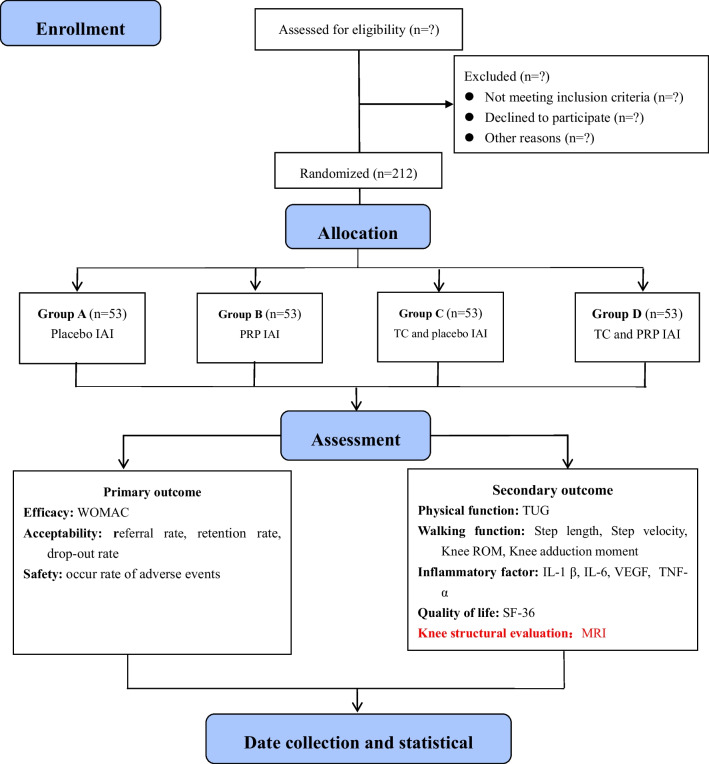
A brief flowchart of the entire study. *IAI* intra-articular injection, *PRP* platelet-rich plasma, *TC* Tai Chi, *WOMAC* Western Ontario and McMaster Universities Osteoarthritis Index, *TUG* Timed Up and Go test, *ROM* Range of Motion, *IL-1 β* Interleukin-1 β,*** IL-6*** Interleukin-6, *VEGF* Vascular Endothelial Growth Factor,* TNF-α* Tumor Necrosis Factor α*;* SF-36 36-Item Short Form Health Survey, *MRI* Magnetic Resonance Image

This protocol will be a placebo-controlled, assessor-blinded randomized trial, which will be included 12-week intervention and 1-year follow-up. This protocol will be conducted in accordance with the Standard Protocol Items: Recommendations for Interventional Trials (SPIRIT) [25]. A total of 212 patients diagnosed with KOA will be randomly and equally assigned into the group A (IAI-placebo), group B (IAI-PRP), group C (TC + IAI-placebo), and group D (TC + IAI-PRP). A brief flowchart of the entire study is shown in Fig. 1, and the schedule of events is provided in Table 1. The study protocol was approved by the Ethics Committee of Shanghai Seventh People’s Hospital (2022-7th-HIRB-067) and registered in the Chinese Clinical Trial Registry (ChiCTR2300067559).

**Table 1:**
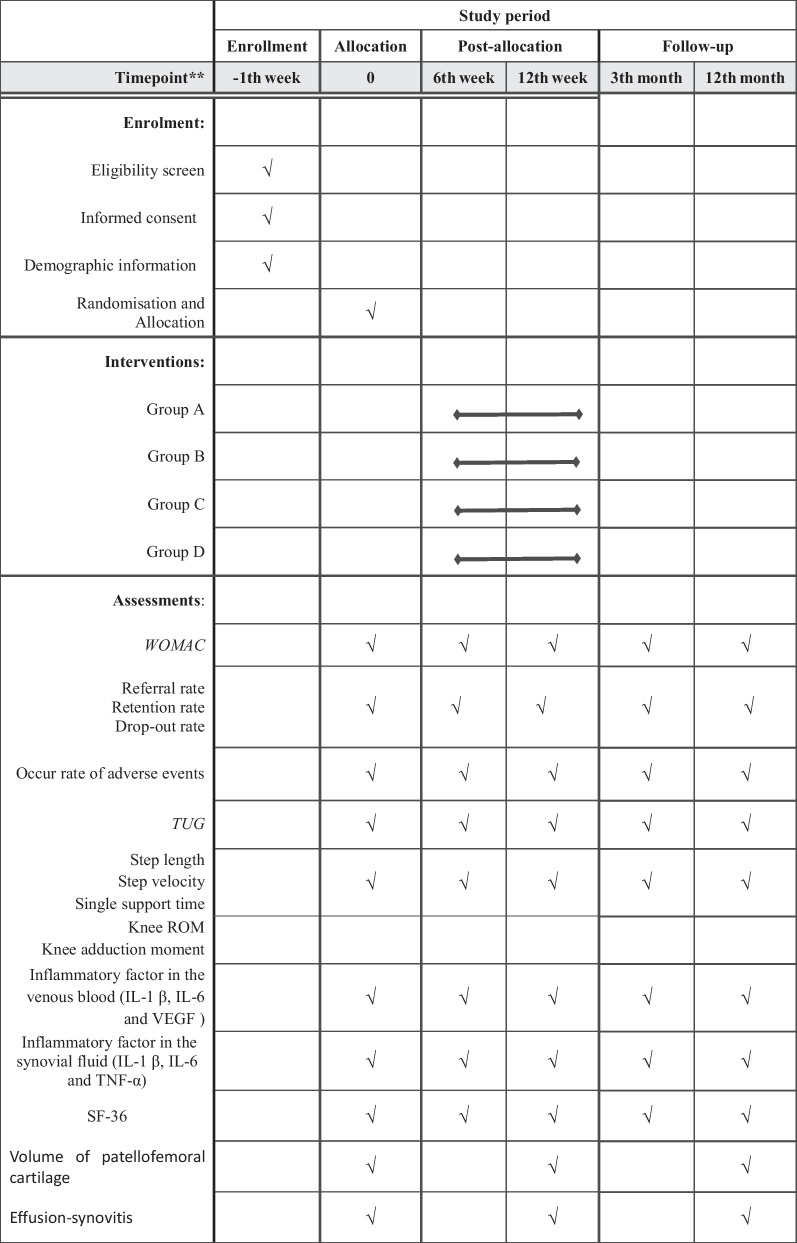
Schedule of enrollment, intervention and assessments.

“√” means things will be done. *WOMAC* Western Ontario and McMaster Universities Osteoarthritis Index, *TUG* Timed Up and Go test, *ROM* Range of Motion, *IL-1 β* Interleukin-1 β,*** IL-6*** Interleukin-6, *VEGF* Vascular Endothelial Growth Factor,*** TNF-α*** Tumor Necrosis Factor α,** SF-36** 36-Item Short Form Health Survey

### Sample size

The sample size will be determined based on the priori power analysis through G*Power software version 3.1.9.2 [26]. In the calculations, ANOVA repeated measures between factor will be chosen, effect size 0.25, alpha 0.05, power 0.80, number of groups 4, number of measures 5, and the sample size is calculated as 168. Taking into account a conservative anticipation of a 20% drop-out rate, the final sample size will therefore be 53 in each group, with a total of 212 subjects.

### Participants

The participants must fulfill the KOA diagnosis criteria of the American College of Rheumatology, including MRI or CT radiography and current joint symptoms based on self-report [27]. The participants will be further screened according to the inclusion and exclusion criteria for those who meet the diagnostic criteria.

### Inclusion criteria

The inclusion criteria will be as follows: (1) meet the diagnostic criteria for KOA; (2) aged 50–75 years; (3) Kellgren–Lawrence (KL) grade I to III based on radiographic classification; (4) without the experience of TC; (5) volunteer to take part in the study and sign the informed consent form.

### Exclusion criteria

The exclusion criteria will be as follows: (1) have the history of trauma, ligament damage, fracture, or surgery on the knee within 12 months, causing pain or functional problems; (2) have severe cardiovascular disease, damage of liver and kidney function, immune deficiency, diabetes mellitus, and blood diseases; (3) have other acute or chronic disorders or psychiatric conditions that will affect physical or cognitive functions; (4) have received medication or relevant intra-articular injection within the last 3 months; (5) have involved in any study related to exercise program within the last 6 months.

### Recruitment and enrollment

Two hundred and twelve participants with KOA will be recruited from the Shanghai Seventh People’s Hospital and neighboring communities, through the ways of flyers, posters, recommended by rehabilitation therapist or neurologist, and WeChat official account. We will communicate with potential participants with participation intention by telephone for the first step of screening and asked them to self-report whether they met the inclusion criteria. For the subjects who passed the first step of telephone screening, he or she will be invited to face-to-face examination and evaluation, and to fully determine that the participants meet the inclusion criteria and not meet the exclusion criteria. In addition, we will inform the purpose, method and potential benefits of the present study to all participants, as well as the principle of voluntary participation. All patients who met the inclusion criteria and agreement to participate will be required to provide their written informed consent.

Recruitment will start from February 1, 2024, until when 212 participants are enrolled.

### Randomization, allocation concealment and blinding

The stratified randomization with gender and KL grade as factors will be used to randomly assign the eligible participants to four groups. First, the participants will be grouped by gender. Then, male and female will be grouped by KL grade. After grouping, there will be six subgroups: (1) male, KL = I; (2) male, KL = II; (3) male, KL = III; (4) female, KL = I; (5) female, KL = II; (6) female, KL = III. For any one subgroup, the participants will randomly divide into group A, B, C and D group, and all group A, B, C and D group are merged to form a new group A, B, C and D group. The randomization number lists will be generated by the random number generator of the SPSS software (Version 22.0; IBM Corp., NY, USA).

The allocation of participants will be hidden in opaque numbered and sequentially sealed envelopes, prepared by a researcher who did not participate in the recruitment and assignment of the groups. The corresponding envelopes will be opened to determine a patient’s group assignment once the participants enrolled have completed all baseline assessments. In addition, physiotherapists with same level, TC coaches and IAI operators also will be followed randomization and randomly assigned to any group.

Due to the visible nature of the exercise intervention, blinding of the study investigators and the participants for TC will be impossible [28]. To ensure blinding of the PRP and concealment of randomization, the independent laboratory pharmacist prepared a syringe with PRP and a syringe with placebo (isotonic saline: 0.9% sodium chloride) [29]. They covered study syringes with a specially manufactured thick plastic covering sheath to conceal the appearance of the study intervention. After the intra-articular injection, the syringe covered by the sheath (containing either the remnants of the PRP or saline) will be handed back to the independent research assistant, who disposed of the syringe in effort to maintain blinding of the study team. The success of blinding will be assessed by asking patients just after the injections what treatment they thought they had received. Furthermore, blinding will be applied to the outcome assessors and statisticians who conduct the final statistical analyses in this protocol to avoid potential detection bias. The outcome assessors will be responsible for data collection and analysis, and they will not be involved in the subject recruitment process.

### Interventions

All participants will receive health education recommended by the guidelines of Osteoarthritis Research Society International (OARSI) [30]. On the basis of health education, the group A will receive the placebo intra-articular injection; the group B will receive the PRP intra-articular injection; the group C will receive TC and placebo intra-articular injection; the group D will receive TC and PRP intra-articular injection. During 12-week intervention, intra-articular injection of PRP and placebo will be performed under ultrasound guidance. The frequency and dose of intra-articular injection will be consistent between the placebo and PRP in the four groups. Injection will be performed once a week, three consecutive times as a course, after a week of rest to continue the next course, a total of 3 courses (12 week). Additionally, the TC interventions in the Group C and D will be carried out 3 days per week for a total of 12 weeks.

### Health education

The efficacy of health education for treat KOA have been demonstrated by the organizations of OARSI and Osteoarthritis and Musculoskeletal Diseases (ESCEO) [30]. Thus, we will use health education as basis intervention. The health education will include two parts: (1) an educational booklet and a video will be provided to the patients to accomplish the home-based self-learning; (2) weekly group meeting with face to face. The topics covered by health education include: osteoarthritis, aging, how to exercise for the elderly, the effect of TC on health, and the education and management of KOA pathogenesis factors, etc.

### PRP preparation and injection

The fresh venous blood samples of the participants will be collected via venipuncture, which underwent the process of two-step differential centrifugation in order to obtain PRP [1, 31]. One syringe of 36 mL of autologous blood will be collected from the median cubital vein, and inject into four 9-mL extraction tubes. Among four extraction tubes, three tubes containing 3.8% trisodium citrate as anticoagulant, and the remaining one contains nothing, which will be used for hematological analysis. After blood collection, the tubes will be submitted to double centrifugation (Smart Prep III, Harvest Technologies Corp, USA). Blood samples will be centrifuged for 10 min at 1800 rpm to separate the erythrocyte layer. The upper plasma layer will be carefully collected in a new sterile propylene tube while attempting to not remove the leukocyte layer. The plasma from all tubes will be centrifuged again for 12 min at 3400 rpm to obtain a two-part plasma, with the upper part consisting of platelet-poor plasma and the lower part consisting of LP-PRP. After discarding platelet-poor plasma, about 5 mL of LP-PRP will eventually be obtained. The product monograph reported that an 1 mL of LP-PRP prepared this method with approximately 1244 × 10^6^ platelets and 24.7 × 10^6^ white blood cells [32]. Finally, approximately 6220 × 10^6^ platelets will be injected into the knee of the patients with KOA under ultrasound guidance.

The patient will be instructed to remain in a supine position, and the knee joint will be slightly flexed. The outer and upper part of the patella of the affected knee will be taken as the injection point, where routine disinfection will be performed and a sterile towel will be spread. A 21-gauge needle will be inserted into the knee joint at the upper outer quadrant of the patella under ultrasonographic guidance, and 5 mL PRP will be injected into the joint cavity after the relevant joint fluid was pumped back. After the injection, patients will be advised to avoid heavy or repetitive stress to the knee joint for 48 h. Patients will be instructed to avoid co-interventions and nonsteroidal anti-inflammatory drugs (NSAIDs) 24 h prior to the intervention [33].

### Tai Chi

The Tai Chi program is mainly based on a nine-form Yang-style, which is a brief and modified version of 64-form Yang-style [34, 35]. It can be easily learned and practiced without restrictions on time and space. More importantly, it is suitable for the patients with KOA, because some movement patterns with high knee joint load are modified by the Tai Chi master to accommodate KOA symptoms and help limit dropouts. The patients will participate in three 60-min Tai Chi sessions conducted weekly for 12 weeks. Each session includes: (1) warm up and review of Tai Chi principles and techniques, 10 min; (2) Tai Chi exercises, 40 min; (3) cool-down relaxation, 10 min. The teaching is carried out by a Tai Chi master who has over 10 years’ experience conducting Tai Chi exercise. Information about the principles and skillsets of each form of TC, and Video clips will be provided to each participant for home learning.

### Outcome measures

Outcome measures will be assessed by profession rehabilitation assessor, biochemical analyst and ultrasound doctor at baseline (T1) before intervention, 6 weeks (T2) and 12 weeks (T3) during intervention, and 3 months (T4) and 12 months (T5) during follow-up period. The demographic and clinical characteristics of the all participants will be collected at the time of enrollment.

### Primary outcomes

1. Efficacy: The Western Ontario and McMaster Universities Osteoarthritis Index (WOMAC) will be used to evaluate the score of the pain, stiffness and daily functional activities of the participants. A number of studies demonstrated that the WOMAC is a reliable and valid instrument for evaluating the joint function of KOA [36, 37]. The aggregate score of the WOMAC is 96, which mainly includes pain (20 points), stiffness (8 points) and daily functional activities (68 points). The higher the score is, the more serious the knee joint dysfunction is. In addition, the anchor-based method will be utilized to determine the minimal clinically important difference (MCID) in order to evaluate the clinical significance of the changes in WOMAC [38]. All patients will be asked to rate their knee improvement compared to baseline at 3 and 12 months. The question will be: “How is your health today compared with baseline?” Patients will be able to choose from five Likert response options: “a great deal better,” “somewhat better,” “equal,” “somewhat worse,” and “a great deal worse.” The MCID will be determined by calculating the average change score for patients who reported their knee improvement as “somewhat better” in the transitional question. This will involve subtracting the baseline scores from the scores at 3 and 12 months during the follow-up period. The MCID proportion (MCID%) will be calculated as the percentage of the sample with a change in scores that surpasses the MCID threshold.
2. Acceptability: The acceptability is an important part of the evaluation of intervention. The rate of referral, retention and drop-out will be used to comprehensively evaluate the acceptability of interventions. • Referral rate is defined as the ratio of the number of referrals made by different departments and hospitals to all participants (T1); • Retention rate is the ratio of the number of participants who completed the study (T5) to all participants (T1); • Drop-out rate is the ratio of the number of participants who dropped out after randomization to all participants (T1), talk with the drop-out participants to identify their reasons for dropping out.
3. Safety: The occur rate of adverse events is defined as the ratio of the number of occurred participants to all participants (T1), which will be used to evaluate the safety of intervention. During the intervention and follow-up, the adverse events (e.g., pain, fall) will be recorded on the Case Report Form (CRF) by the way of the monitor and self-report, and will be evaluated for relevance to the intervention.

### Secondary outcomes

1. Physical function: The Timed Up and Go test (TUG) will be used to assess the physical function performance of the participants. Previous study reported that the TUG is simple, reliable and valid measurement for assess the physical performance of the KOA [39, 40]. During TUG test, the participants will be required to stand up from a standard chair (40 cm height), walk 3 m, turn around, walk back, and sit down again. The test will be performed 3 times and record with chronograph.
2. Walking function: The gait analysis of the self-selected speed walking will be used to assess the walking function for KOA. The gait analysis is chosen as functional measures of walking, due to the quantitative and systematic evaluation methods, could more comprehensively assess the changes of walking function than the graded assessment scale [41, 42]. Such as, the knee adduction moment can effectively reflect the degree of pressure imbalance between the medial and lateral compartments of the knee joint [42]. Full body three-dimensional kinematics and ground reaction forces are collected by a 10-camera infrared motion capture system (Vicon Motion Systems, Oxford, United Kingdom) and the force platform (Kistler Instruments AG Corp., Switzerland), respectively. The human biomechanical model is built based on Visual 3D (C-Motion, Inc., United States). The gait analysis outcomes will be included: temporospatial parameters (e.g., step length, step velocity, single support time), the range of motion and knee adduction moment.
3. Inflammatory factor levels: The fasting venous blood, synovial fluid of the knee joint will be collected from the patients for analysis the inflammatory factor levels [43, 44]. • The inflammatory factor in the venous blood: 5 ml of fasting venous blood will be collected from the patients in the morning with routine local disinfection. The Interleukin-1 β (IL-1 β), interleukin-6 (IL-6), and vascular endothelial growth factor (VEGF) will be measured by Enzyme-Linked Immunosorbent Assay (ELISA). Furthermore, erythrocyte sedimentation rate (ESR) was measured by Westergren method. • The inflammatory factor in the synovial fluid: 2 mL of synovial fluid will be withdrawn from the joint cavity by puncturing the syringe just above the patella and lateral quadriceps femoris. For those who could not be extracted successfully due to the lack of joint fluid, 0.9% chlorinated steel injection (2 mL) will be injected into the joint cavity to move the knee joint and then extracted [43]. The IL-1 β, IL-6 and tumor necrosis factor α (TNF-α) will be measured by ELISA.
4. Quality of life: The 36-Item Short Form Health Survey (SF-36) will be used to comprehensively evaluate the quality of life across broad physical and emotional health domains of the participants. Previous study reported that the SF-36 is easy to use, acceptable to patients, and fulfills stringent criteria of reliability and validity [45, 46]. The SF-36 questionnaire includes eight multiple-item subscales, the score on each SF-36 subscale ranges between 0 and 100, and greater score indicates better quality of life.
5. Knee structural evaluation: The knee structural changes will be evaluated by 3 Tesla Philips MRI scanners and a sense coil for the following sequences: transverse 3D TRUFISP and coronal and sagittal fat saturated protondensity. • Volume of patellofemoral cartilage: The cartilage area was manually segmented from the nearby bone and soft tissue in the TRUFISP 3D sequence. Subsequently, the volume of the cartilage was determined by multiplying the sum of the cartilage area in all the images by the slide thickness, utilizing 3D slicer software [47]. • Effusion-synovitis: The presence of intra-articular fluid-equivalent signal on the proton density-weighted images was used to define effusion-synovitis [48]. A semi-automated segmentation technique was employed to measure the volume of effusion-synovitis. The final 3D volume rendering was created using 3D Slicer, a free open-source imaging software (version 4.10, National Alliance of Medical Image Computing, NA-MIC) [49].

### Data collection and management

The demographic, clinical characteristics, outcome measures, adverse events, and safety evaluations will be collected and recorded in the CRF. The outcome assessor fill-in relevant information timely and accurately according to the CRF requirement. The data check (e.g., checked against the CRF of the origin data, double-checking) will be conducted to ensure the data accuracy before electronic storage. The two research assistants who are blinded the study process will enter all the collected data into the data management system with a double entry method. In addition, they also will enter the real-time data into the Chinese Clinical Trial Registration Center. Access to the dataset will be restricted to the Clinical Trial Management and the Data Safety and Monitoring Board. Storage and disposal of research data hard copies will strictly follow the regulations and policies of the investigator’s institution and the study sites. In addition, serious adverse events will be reported to an independent Data Safety and Monitoring Board that will advise whether to continue, modify or stop the intervention. During the study process, to protect individual privacy before, the dataset will be stored, analyzed and archived in a pseudonymized manner.

### Statistical analysis

All statistical analyses will be performed with IBM SPSS (Version 22.0, Chicago, IL, USA) by statisticians who are blinded to the group allocation. The outcomes analyses will be performed on an intention-to-treat basis. For the missing data, several sensitivity analyses will be conducted to evaluate their effects on the results. The Shapiro–Wilk will be used to test the normal distribution of the continuous variables for demographic and outcome measures. Continuous variables will be described as mean ± SD for normal distributions or median for non-normal distributions, categorical variables will be described as frequency. The χ^2^ test or Fisher’s exact test will be used to examine the comparisons between the four groups for categorical variables. When the normality of data distribution is found, two-way of variance with repeated measures will be applied to examine the main effects of the group and the time factor and group-time interaction effects. A simple effect post hoc analysis will be conducted, when time-group interaction is significant. Additionally, if homogeneity is not found, a linear mixed model will be adjusted for gender and KL grade. To mitigate bias and potential confounding variables, the propensity score matching (PSM) method will be employed [50]. Significant level for all tests will be set at 0.05 for all statistical tests and corrected for multiple comparisons using the Bonferroni-adjusted method, and 95% confidence interval will be reported.

### Patient and public involvement

The initial study idea was conceived by the study team. The patients with KOA, physiotherapists and neurologist took part in preparation of the proposal with face-to-face interviews. In addition, the study protocols also will be modified and supplemented utter concerns not addressed in a draft proposed at the time based on their feedback, to ensure the safety and applicability of the intervention.

### Dissemination plans

The results of this study will be fully published in international peer-reviewed journals, with both positive and negative results reported.

## Discussion

The systematic review reported that no treatment methods have significant modifying properties and long-term efficacy for KOA [2, 11]. The development of an ideal and effective programs for treatment of KOA is still being explored. The pathological mechanisms of KOA involved the progress of the biochemical and biomechanical [8, 51], but no integrated treatment programs for the aspects of the biomedical and biomechanical mechanisms. In our knowledge, this trial is first one to propose an integrated scheme combing IAI-PRP and TC for treatment of KOA, based on the consideration of the biochemical and biomechanical pathogenesis of KOA. Furthermore, the efficacy, acceptability and safety of the combined IAI-PRP and TC will be explored by the design of 12-week intervention and 12-month follow-up. These results of the study will provide evidence with high quality for integrated IAI-PRP and TC, and guide significance for design more effective treatment methods in KOA.

This study has several important strengths. First, it is a novel integrated treatment method for combing PRP IAI and TC interventions for KOA. Previous study examined individual components (e.g., health education, PRP, TC) but have not combined them in an integrated method [16, 22, 52]. More importantly, this method takes into account the biochemical and biomechanical factors in the development of KOA, and may have a potential opportunity to treat KOA. Second, the comprehensively intervention protocol will be evidence-based and rigorously developed based on the evidence, recommendations, theories and practice standards of the systematic review [30, 40, 53], and will be allowed to achieve multiple study purpose such as examine the effectiveness and safety of combined IAI-PRP and TC for KOA treatment and compare the differences of efficacy between the IAI-PRP and TC in the KOA treatment. The questions will be answered by these study purposes which are also the problems that the need to be solved in the field of KOA treatment in the systematic review wrote by Cook [6]. Third, Medical Research Council Framework for developing and evaluating complex interventions suggested that the feasibility, acceptability and safety of a proposed intervention and research methodological procedures should be fully examined prior to performing the full-scale study [54, 55]. The comprehensive assessment of efficacy, acceptability and safety was designed in this study, such as drop-out rate will be reflected acceptability and adverse events occur rate will be reflected safety. Last, more systematic and comprehensive assessment outcomes will be selected in this protocol, such as knee adduction moment from 3D gait analysis, inflammatory factor levels from venous blood and synovial fluid. Therefore, the present study will provide more comprehensive and systematic protocol for the further study with randomized controlled.

We recognize this study also has limitations. This research has an inevitable limitation associated with the difficulty in controlling the methodology of blinding during the TC intervention, which cannot be blinded for the participants and therapists due to the visible of the TC intervention. Although the outcome assessors and data analyzer will be blinded to the group allocation, which might increase the risk of detection bias during the study’s implementation. Additionally, the PRP preparation and injection need to be operated by medical personnel in medical institutions, which may limit the promotion and application of the combined IAI-PRP and TC in the community and home.

In summary, the results of the study protocol will demonstrate that the efficacy, acceptability and safety of the combined IAI-PRP and TC for treatment of KOA. This method may have a potential opportunity to treat KOA. The study protocol will provide guide significance in integrate exist and different rehabilitation technology and design more effective rehabilitation program for KOA.

## Data Availability

Not applicable.

## Abbreviations

KOA: Knee osteoarthritis
IAI: Intra-articular injection
PRP: Platelet-rich plasma
HA: Hyaluronic acid
MSCs: Mesenchymal stem cells
TC: Tai Chi
MRI: Magnetic resonance image
CT: Computed tomography
KL: Kellgren–Lawrence
OARSI: Osteoarthritis Research Society International
ESCEO: Osteoarthritis and Musculoskeletal Diseases
CBCs: Complete blood counts
WOMAC: Western Ontario and McMaster Universities Osteoarthritis Index
CRF: Case report form
TUG: Timed up and go test
IL-1 β: Interleukin-1 β
IL-6: Interleukin-6
VEGF: Vascular endothelial growth factor
ELISA: Enzyme-linked immunosorbent assay
TNF-α: Tumor necrosis factor α
SF-36: 36-Item short form health survey

## References

[1] Ma Q, Hei X, Zhu S, Tian X, Chen Y, Zhu N, Zhang J, Feng P, Liu Y, Xing L (2022). The clinical efficacy of Platelet-Rich Plasma versus conventional drug injection in the treatment of knee osteoarthritis: a study protocol for a randomized controlled trial. Evid Based Complement Alternat Med.

[2] Cai Z, Cui Y, Wang J, Qi X, He P, Bu P, Xu Y, Li Y (2022). A narrative review of the progress in the treatment of knee osteoarthritis. Ann Transl Med.

[3] Cui A, Li H, Wang D, Zhong J, Chen Y, Lu H (2020). Global, regional prevalence, incidence and risk factors of knee osteoarthritis in population-based studies. EClinicalMedicine.

[4] Aw AAL, Leeu JJ, Tao X (2021). Bin Abd Razak HR: Comparing the efficacy of dual Platelet-Rich Plasma (PRP) and Hyaluronic Acid (HA) therapy with PRP-alone therapy in the treatment of knee osteoarthritis: a systematic review and meta-analysis. J Exp Orthop.

[5] Phillips M, Bhandari M, Grant J, Bedi A, Trojian T, Johnson A, Schemitsch E (2021). A systematic review of current clinical practice guidelines on intra-articular Hyaluronic Acid, Corticosteroid, and Platelet-Rich Plasma injection for knee osteoarthritis: an international perspective. Orthop J Sports Med.

[6] Cook CS, Smith PA (2018). Clinical update: why PRP should be your first choice for injection therapy in treating osteoarthritis of the knee. Curr Rev Musculoskelet Med.

[7] Agarwal BM, Yadav RP, Tambe SD, Kulkarni CC, Mullerpatan RP (2021). Evaluation of early knee osteoarthritis using biomechanical and biochemical markers. Crit Rev Biomed Eng.

[8] Teichtahl AJ, Wluka AE, Wijethilake P, Wang Y (2015). Wolff's law in action: a mechanism for early knee osteoarthritis. Arthritis Res Ther.

[9] van Tunen JA, Dell'Isola A, Juhl C, Dekker J, Steultjens M, Lund H (2016). Biomechanical factors associated with the development of tibiofemoral knee osteoarthritis: Protocol for a systematic review and meta-analysis. BMJ Open.

[10] Moussa M, Lajeunesse D, Hilal G, El Atat O, Haykal G, Serhal R, Chalhoub A, Khalil C, Alaaeddine N (2017). Platelet rich plasma (PRP) induces chondroprotection via increasing autophagy, anti-inflammatory markers, and decreasing apoptosis in human osteoarthritic cartilage. Exp Cell Res.

[11] Brody LT (2015). Knee osteoarthritis: clinical connections to articular cartilage structure and function. Phys Ther Sport.

[12] Georgiev T, Angelov AK (2019). Modifiable risk factors in knee osteoarthritis: treatment implications. Rheumatol Int.

[13] Liao CD, Chen HC, Huang MH, Liou TH, Lin CL, Huang SW (2023). Comparative efficacy of Intra-articular injection, physical therapy, and combined treatments on pain, function, and sarcopenia indices in knee osteoarthritis: a network Meta-analysis of randomized controlled trials. Int J Mol Sci.

[14] Zhao J, Huang H, Liang G, Zeng LF, Yang W, Liu J (2020). Effects and safety of the combination of platelet-rich plasma (PRP) and hyaluronic acid (HA) in the treatment of knee osteoarthritis: a systematic review and meta-analysis. BMC Musculoskelet Disord.

[15] Sit RWS, Wu RWK, Law SW, Zhang DD, Yip BHK, Ip AKK, Rabago D, Reeves KD, Wong SYS (2019). Intra-articular and extra-articular platelet-rich plasma injections for knee osteoarthritis: a 26-week, single-arm, pilot feasibility study. Knee.

[16] Dório M, Pereira RMR, Luz AGB, Deveza LA, de Oliveira RM, Fuller R (2021). Efficacy of platelet-rich plasma and plasma for symptomatic treatment of knee osteoarthritis: a double-blinded placebo-controlled randomized clinical trial. BMC Musculoskelet Disord.

[17] Andia I, Maffulli N (2018). Some patients (and some of us) respond better to some biological therapies: the as yet unsolved conundrum. J Orthop Traumatol.

[18] Kaszyński J, Bąkowski P, Kiedrowski B, Stołowski Ł, Wasilewska-Burczyk A, Grzywacz K, Piontek T (2022). Intra-articular injections of autologous adipose tissue or Platelet-Rich Plasma comparably improve clinical and functional outcomes in patients with knee osteoarthritis. Biomedicines.

[19] Raeissadat SA, Rayegani SM, Hassanabadi H, Fathi M, Ghorbani E, Babaee M, Azma K (2015). Knee osteoarthritis injection choices: Platelet-Rich Plasma (PRP) versus Hyaluronic Acid (A one-year randomized clinical trial). Clin Med Insights Arthritis Musculoskelet Disord.

[20] Paterson KL, Nicholls M, Bennell KL, Bates D (2016). Intra-articular injection of photo-activated platelet-rich plasma in patients with knee osteoarthritis: a double-blind, randomized controlled pilot study. BMC Musculoskelet Disord.

[21] Hu L, Wang Y, Liu X, Ji X, Ma Y, Man S, Hu Z, Cheng J, Huang F (2021). Tai Chi exercise can ameliorate physical and mental health of patients with knee osteoarthritis: systematic review and meta-analysis. Clin Rehabil.

[22] You Y, Liu J, Tang M, Wang D, Ma X (2021). Effects of Tai Chi exercise on improving walking function and posture control in elderly patients with knee osteoarthritis: a systematic review and meta-analysis. Medicine (Baltimore).

[23] Kolasinski SL, Neogi T, Hochberg MC, Oatis C, Guyatt G, Block J, Callahan L, Copenhaver C, Dodge C, Felson D (2020). 2019 American College of Rheumatology/Arthritis Foundation guideline for the management of osteoarthritis of the hand, hip, and knee. Arthritis Care Res (Hoboken).

[24] Ye J, Cai S, Zhong W, Cai S, Zheng Q (2014). Effects of tai chi for patients with knee osteoarthritis: a systematic review. J Phys Ther Sci.

[25] Chan AW, Tetzlaff JM, Gotzsche PC, Altman DG, Mann H, Berlin JA, Dickersin K, Hrobjartsson A, Schulz KF, Parulekar WR (2013). SPIRIT 2013 explanation and elaboration: guidance for protocols of clinical trials. BMJ.

[26] Yang S, Chen J, Guo Y, Teng Y, Liu T, Ying R, He Z, Wu J, Yu SG, Zeng F (2019). Comparison of Taiji and aerobic exercise for functional constipation: study protocol for a randomised controlled neuroimaging trial. BMJ Open.

[27] Kolasinski SL, Neogi T, Hochberg MC, Oatis C, Guyatt G, Block J, Callahan L, Copenhaver C, Dodge C, Felson D (2020). 2019 American College of Rheumatology/Arthritis Foundation Guideline for the Management of Osteoarthritis of the Hand, Hip, and Knee. Arthritis Rheumatol.

[28] Guo G, Kong Y, Zhu Q, Wu Z, Zhang S, Sun W, Cheng Y, Fang M (2022). Cerebral mechanism of Tuina analgesia in management of knee osteoarthritis using multimodal MRI: study protocol for a randomised controlled trial. Trials.

[29] Paget LDA, Reurink G, de Vos RJ, Weir A, Moen MH, Bierma-Zeinstra SMA, Stufkens SAS, Kerkhoffs G, Tol JL, Group PS. Effect of platelet-rich plasma injections vs placebo on ankle symptoms and function in patients with ankle osteoarthritis: a randomized clinical trial. Jama 2021;326(16):1595–1605 10.1001/jama.2021.16602 PMC8548954 34698782

[30] Arden NK, Perry TA, Bannuru RR, Bruyère O, Cooper C, Haugen IK, Hochberg MC, McAlindon TE, Mobasheri A, Reginster JY (2021). Non-surgical management of knee osteoarthritis: comparison of ESCEO and OARSI 2019 guidelines. Nat Rev Rheumatol.

[31] Zhao JL, Huang HT, Liang GH, Zeng LF, Yang WY, Liu J (2020). Effects and safety of the combination of platelet-rich plasma (PRP) and hyaluronic acid (HA) in the treatment of knee osteoarthritis: a systematic review and meta-analysis. Bmc Musculoskel Dis.

[32] Gilat R, Haunschild ED, Knapik DM, Evuarherhe A, Parvaresh KC, Cole BJ (2021). Hyaluronic acid and platelet-rich plasma for the management of knee osteoarthritis. Int Orthop.

[33] Gupta A, Jeyaraman M, Maffulli N (2022). Common medications which should be stopped prior to Platelet-Rich Plasma injection. Biomedicines.

[34] Wang X, Hou M, Chen S, Yu J, Qi D, Zhang Y, Chen B, Xiong F, Fu S, Li Z (2020). Effects of tai chi on postural control during dual-task stair negotiation in knee osteoarthritis: A randomised controlled trial protocol. BMJ Open.

[35] Song J, Wei L, Cheng K, Lin Q, Xia P, Wang X, Wang X, Yang T, Chen B, Ding A (2022). The Effect of modified Tai Chi exercises on the physical function and quality of life in elderly women with knee osteoarthritis. Front Aging Neurosci.

[36] Angst F, Ewert T, Lehmann S, Aeschlimann A, Stucki G (2005). The factor subdimensions of the Western Ontario and McMaster Universities Osteoarthritis Index (WOMAC) help to specify hip and knee osteoarthritis. A prospective evaluation and validation study. J Rheumatol.

[37] Copsey B, Thompson JY, Vadher K, Ali U, Dutton SJ, Fitzpatrick R, Lamb SE, Cook JA (2019). Problems persist in reporting of methods and results for the WOMAC measure in hip and knee osteoarthritis trials. Qual Life Res.

[38] Kim MS, Koh IJ, Choi KY, Sung YG, Park DC, Lee HJ, In Y (2021). The minimal clinically important difference (MCID) for the WOMAC and factors related to achievement of the MCID after medial opening wedge high tibial osteotomy for knee osteoarthritis. Am J Sports Med.

[39] Lai Z, Lee S, Hu X, Wang L (2019). Effect of adding whole-body vibration training to squat training on physical function and muscle strength in individuals with knee osteoarthritis. J Musculoskelet Neuronal Interact.

[40] Shimizu H, Shimoura K, Iijima H, Suzuki Y, Aoyama T (2022). Functional manifestations of early knee osteoarthritis: a systematic review and meta-analysis. Clin Rheumatol.

[41] Gu C, Mao Y, Dong H, Cui Y, Fu M (2022). Nomogram in knee instability: 3D gait analysis of knee osteoarthritis patients. Indian J Orthop.

[42] Khalaj N, Abu Osman NA, Mokhtar AH, Mehdikhani M, Wan Abas WA (2014). Effect of exercise and gait retraining on knee adduction moment in people with knee osteoarthritis. Proc Inst Mech Eng H.

[43] Sun Z, Su W, Wang L, Cheng Z, Yang F (2022). Clinical effect of Bushen Huoxue method combined with Platelet-Rich Plasma in the treatment of knee osteoarthritis and its effect on IL-1, IL-6, VEGF, and PGE-2. J Healthc Eng.

[44] Deek MS, Abry DS, El Seedek H, Arweash A (2021). Intra-articular injection of Platelet rich plasma versus Hyaluronic acid for moderate knee osteoarthritis. A prospective, double-blind randomized controlled trial on 189 patients with follow-up for three years. Acta Orthop Belg.

[45] Brazier JE, Harper R, Jones NM, O'Cathain A, Thomas KJ, Usherwood T, Westlake L (1992). Validating the SF-36 health survey questionnaire: new outcome measure for primary care. BMJ.

[46] Husted JA, Gladman DD, Farewell VT, Long JA, Cook RJ (1997). Validating the SF-36 health survey questionnaire in patients with psoriatic arthritis. J Rheumatol.

[47] Raeissadat SA, Ghorbani E, Sanei Taheri M, Soleimani R, Rayegani SM, Babaee M, Payami S (2020). MRI changes after Platelet Rich Plasma injection in knee osteoarthritis (randomized clinical trial). J Pain Res.

[48] Drummen SJJ, Balogun S, Lahham A, Bennell K, Hinman RS, Callisaya M, Cai G, Otahal P, Winzenberg T, Wang Z (2023). A pilot randomized controlled trial evaluating outdoor community walking for knee osteoarthritis: walk. Clin Rheumatol.

[49] Wang Z, Jones G, Winzenberg T, Cai G, Laslett LL, Aitken D, Hopper I, Singh A, Jones R, Fripp J (2020). Effectiveness of curcuma longa extract for the treatment of symptoms and effusion-synovitis of knee osteoarthritis: a randomized trial. Ann Intern Med.

[50] Kane LT, Fang T, Galetta MS, Goyal DKC, Nicholson KJ, Kepler CK, Vaccaro AR, Schroeder GD (2020). Propensity score matching: a statistical method. Clin Spine Surg.

[51] Herrero-Beaumont G, Roman-Blas JA, Bruyère O, Cooper C, Kanis J, Maggi S, Rizzoli R, Reginster JY (2017). Clinical settings in knee osteoarthritis: Pathophysiology guides treatment. Maturitas.

[52] Chen H, Zheng X, Huang H, Liu C, Wan Q, Shang S (2019). The effects of a home-based exercise intervention on elderly patients with knee osteoarthritis: a quasi-experimental study. BMC Musculoskelet Disord.

[53] Andia I, Maffulli N (2019). Blood-derived products for tissue repair/regeneration. Int J Mol Sci.

[54] De Silva MJ, Breuer E, Lee L, Asher L, Chowdhary N, Lund C, Patel V (2014). Theory of change: a theory-driven approach to enhance the medical research council's framework for complex interventions. Trials.

[55] Andia I, Maffulli N (2018). A contemporary view of platelet-rich plasma therapies: moving toward refined clinical protocols and precise indications. Regen Med.

